# A Dataset of 10,000 Situations for Research in Computational Social Sciences Psychology and the Humanities

**DOI:** 10.1038/s41597-023-02406-6

**Published:** 2023-07-29

**Authors:** Yair Neuman, Yochai Cohen

**Affiliations:** 1 Head, The Functor Lab, Department of Cognitive and Brain Science, Ben-Gurion University of the Negev, Beer-Sheva, 84105 Israel; 2 Gilasio coding, Tel-Aviv, Israel

**Keywords:** Interdisciplinary studies, Human behaviour

## Abstract

It has been realized that situational dimensions, as represented by human beings, are crucial for understanding human behavior. The Riverside Situational Q (RSQ) is a tool that measures the psychological properties of situations. However, the RSQ-4 includes only 90 items and may have limited use for researchers interested in measuring situational dimensions using a computational approach. Here we present a corpus of 10,000 artificially generated situations corresponding mostly with the RSQ-4. The dataset was generated using GPT, the state-of-the-art large language model. The dataset validity is established through inter-judge reliability, and four experiments on large datasets support its quality. The dataset and the code used for generating 100 situational dimensions may be useful for researchers interested in measuring situational dimensions in textual data.

## Background & Summary

“Daily life unfolds in a sequence of situational contexts, which are pivotal for explaining people’s thoughts, feelings, and behaviors”^1^ (p. 1). From a psychological perspective, situations involve how subjects *represent* the context in which they operate. These representations may range from subjective representations characterizing individuals to highly objective representations shared by large collectives. The evolutionary perspective^2^ suggests that beyond cultural and individual variability, the representations of situations may have a common denominator grounded in our basic human experiences. For example, regardless of cultural or individual particularities, representing a situation as “potentially enjoyable” is a level of representation shared by human beings across cultures. Nevertheless, cultural particularity should always be of concern when developing computational tools^3^. However, some situational tools have been tested and validated across cultures^4^, and there seems to be no substantial barrier to developing and using culturally sensitive tools for situational analysis. The current paper has no universal pretensions to provide a dataset applicable to all cultures. We are working on new tools for identifying situational dimensions that may be used for various cultures.

As situations and personality influence behavior^5^, it is important to quantify and measure situational dimensions. The Riverside Situational Q sort (RSQ) has been designed to quantify and measure situational dimensions^6^. The RSQ has been formed in correspondence with the California Adult Q-Sort, where for each of the 100 personality descriptors in the CAQ, the researcher tried to match an aspect of the context that may lead to similar behavior. The recent version of the RSQ^7^ includes 90 items, such as “The situation is potentially enjoyable” and “Someone is criticizing you.”

The importance of measuring situations goes beyond psychology. Researchers in computational social sciences, for instance, may find interest in measuring situational dimensions to support various classification tasks. For example, a recent DARPA project focuses on automatically identifying social norms violations (e.g., Dressing inappropriately for school). In this context, it was shown^8^ that social norm violations can be automatically detected. To decide whether a social norm has been violated, measuring situational dimensions forming the context in which the behavior is analyzed could have been helpful. Another example in which computational social sciences and psychology may benefit from automatic situational analysis is predicting change in emotion during a dialogue^9^. In this context, measuring the situational dimensions for better predicting a change may be helpful. For instance, measuring how the situation changes may help us predict an approaching emotional change.

The dataset and code we provide in this paper may be highly relevant for researchers from various fields. For example, in the study of personality, it was emphasized a long time ago that personality is context-dependent^10^. Therefore, researchers may use modern AI tools for personality analysis combined with the proposed measurements of situational dimensions to better understand the context-dependent nature of personality. For instance, one may analyze teenagers’ social media texts to better understand how the presented personality fluctuates from introvert to extrovert in different situations. Predicting the individual’s personality based on situational features may contribute to our understanding of personality and provide a new methodology for the person-specific paradigm in psychology^11^. Moreover, the dataset and the accompanied code may be of great use to researchers in digital and computational humanities^12^ because the idea of “context” has been central to the humanities. Therefore, the dataset and the code may be used to support humanities researchers with a powerful contextual analysis of their data.

In sum, computational social sciences, psychology, and humanities may benefit from the RSQ. However, the limited number of items is a barrier for many projects requiring a large dataset on which different Machine Learning classifiers can be trained and tested. This paper aims to introduce a large dataset of situations based on the RSQ-4. As our interest in situations includes prestige and dominance in human interactions, we added ten items measuring the extent to which the situation involves *prestige* and *dominance*^13^. These are two important aspects of our social life associated with social rank^14^. The ten items we have designed as prompts for the generation of synthetic items measuring dominance and prestige (see Methods) have been inspired by the dominance-prestige scales^15^.

## Methods

### The dataset construction

We start with 90 items from the RSQ-4, five items measuring prestige in a situation and five items measuring dominance in a situation. Next, we used the state-of-the-art Large Language Model to generate a large dataset of situations. More specifically, we used GPT 3.5^16^ and applied the following template for each of the 100 items (the 90 RSQ-4 items + the 10 prestige-dominance items). The template was used as a prompt for GPT.

### The template

Given items 1 to 100 (e.g., “The situation is potentially enjoyable”).

Use the following prompt:

“I’m a university researcher studying situations. My current interest is in [description of the situation, for example, “potentially enjoyable situations”]. Please generate 100 different [request: e.g., “potentially enjoyable situations”]. Each situation should be no more than 100 words long.

An example of the prompt used to generate examples for item 4 in the RSQ-4 (“Someone is trying to impress you”) is as follows:

I’m a university researcher studying situations. My current interest is in situations where someone is trying to impress you. Please generate 100 different situations where someone is trying to impress you. Each situation should be no more than 100 words long.

Using GPT-3.5, we generated 10,000 situations: one hundred examples for each of the one hundred items composing the basic set. The dataset^17^ is available for researchers.

As the dataset includes synthetic examples, it is important to establish its validity and quality. We took the following steps to support the validity and quality of the dataset. First, we selected a dataset sample and used expert human judgment to establish inter-judge reliability and validity. Second, as the data is synthetic, we adopted a common approach in “hard” computational sciences^18^ to establish the dataset’s quality. We hypothesized that if the situations produced by GPT validly represent the original items (i.e., situations), they can be used for measuring situational dimensions in textual data. More specifically, we used the dataset for training a Machine Learning model that can measure situational dimensions in textual data. Next, we used the measurements produced by the model as input features (i.e., variables) for another ML model. This model (i.e., Boosting classification) was then used for classification tasks on four different datasets. We further hypothesized that if the dataset validly represents situational dimensions, then the ML model trained on this dataset can be useful for various tasks in which measuring the situational dimensions of the text may be important. We test this hypothesis in four different experiments.

### Measuring inter-judge reliability

Two students who gained one year of expertise in text analysis analyzed a sample of 500 items. The instructions were: “In the following spreadsheet, you will observe a list of 100 situations. To the right of each situation, you will see five examples illustrating this situation. For each example, please determine whether the example represents the situation (mark “1”) or not (mark “0”).” The first Judge (“L) agreed in 97.6% of the cases that the examples represent the situation. The second Judge (“A”) agreed in 95% of the cases that the examples represent the situation. On average, the judges agreed with the synthetic examples in 96% of the cases. This result supports the validity of the dataset. The association between the ratings of the two judges was statistically significant (χ^2^ = 34.80, p < 0.001). As *Kappa* coefficients “performed best across variations in characteristic prevalence and raters’ expertise and bias^19^” (p. 1), we also measured the inter-rater reliability using Fleiss Multirater Kappa that was also found to be statistically significant (Kappa = 0.242, p < 0.001).

### Measuring the situational dimensions

For each of the 100 situations, we trained Setfit^20,21^ for multiclass classification on 20% of the set (N = 20) and tested the classifier’s performance on the remaining 80%. On average, the classifier gained 85% accuracy, 85% precision, 86% recall, and an f1 score of 0.85. We explain the meaning of these performance measures in the technical validation section.

The procedure introduced so far provides us with a dataset of 10,000 situations and a trained model that generates 100 measurements (i.e., dimensions, features, variables) for each input text. Each measurement represents the extent to which the situational dimension is represented in a text. The Setfit model is available for use. Next, we used the model to measure situational dimensions in texts, fed these measurements into another classifier, and used them for classification tasks.

## Data Records

The Data Records are published in Figshare: 10.6084/m9.figshare.23254856.v2^17^.

All files are CSV files with a very simple structure. The files are:

1. Situations_Dataset: This file includes 10,000 situations. The first column is the Running index; the Second column is the running index of the original situations; the Third column is the running index of the generated examples; and the Fourth column: The example generated by GPT.
  The results files for the 4 experiments are as follows:
2. EMPDIAL_EXP1
3. POLIT_EXP2
4. MORALSTO_EXP3
5. SOCHEM_EXP4

The columns in each file include the measurements of the 100 dimensions (i.e., Funder 1 to 100) and the predicted label.

## Technical Validation

The technical validation section includes four experiments that aimed to support the dataset’s quality. The validation process was the same for all experiments. First, we used a dataset of labeled texts. For instance, in the first experiment, we used a dataset with texts labeled as either representing the emotions associated with adherence to social norms (e.g., pride) or with emotions associated with violating social norms (e.g., shame). Second, we measured the situational dimensions in each text and used these measurements as input to a Machine Learning classifier that aimed to classify the texts into one of the classes (e.g., norm adherence vs. norm violation). We describe the dataset for each experiment with its labels, justification, and data analysis and results. In sum, we measured the dataset’s quality by measuring the 100 dimensions for each text and using these dimensions for classification. We hypothesized that if the situations are valuable, then automatically measuring them in texts may be successfully used for classifying the texts into pre-identified classes which are theoretically grounded.

We use three performance measures for each experiment: accuracy, precision, and recall. To explain the performance measures, we use a toy example. Suppose we test our dataset by using 100 labeled movie plots. Half of the plots describe movies belonging to the genre of Romantic Comedy, and the other half belongs to the genre of Horror. We hypothesize that by measuring the situational dimensions of the plots, we can automatically classify the genre as either Romantic Comedy or Horror. We measure the 100 dimensions for each plot and feed them into a Machine Learning classifier that uses 80% of the cases for learning a model and the rest 20% for tests. It means that we test the Machine Learning model on 20 movie plots. During the test phase, Machine Learning is exposed to 20 plots and asked to classify them as either Romantic Comedy or Horror. The imaginary performance of the Machine Learning classifier is presented Table 1.

**Table 1:** Imaginary performance of the classifier.

Decision	Label	
	Romantic Comedy	Horror
Romantic Comedy	9	2
Horror	1	8

The Decision column describes the classifier’s decision on whether the plot is a Romantic Comedy or Horror. We crosstab the decision against the real label of the plot and whether it is a Romantic Comedy or Horror film. We have 20 plots: ten plots labeled Romantic Comedy and ten labeled Horror. Our first measure is Accuracy: The percentage of cases correctly labeled as Romantic Comedy or Horror. We can see that 17 out of 20 cases were correctly identified; therefore, our classifier’s accuracy is 85%. Now for the Recall performance measure. We may first ask what percent of the cases labeled as Romantic Comedy the classifier correctly identified. We can see that the classifier identified 9 out of 10 cases. Therefore, the recall is 90%. Next, we ask the following question: In how many cases the classifier correctly labeled the case as Romantic Comedy? We can see that the classifier labeled 11 cases as Romantic Comedy and was correct in 9 of them. Therefore, the Precision of the classifier is 82%. Are these good performances? One way to answer this question is to compare the performance to our baseline prediction. We know that 50% of the cases are labeled “Romantic Comedy.” In this case, correctly identifying 90% of the cases, and with 82% Precision, may be considered a significant improvement in prediction over the baseline. In this paper, we use huge datasets, and the performance measures’ contribution to the prediction can be better appreciated.

### The Experiments

#### Experiment 1

We first used the EmpatheticDialogues dataset^22^. This dataset of 25k conversations is grounded in situations, each labeled according to one of 32 emotion labels. Adopting the approach used in another paper^8^, we selected situations labeled by the social emotions of embarrassment, shame, and guilt (N = 4731, labeled as 1) as involving social norm violation. We compared them to situations labeled with pride or gratitude (N = 3621, labeled as 0) representing adherence to social norms.

##### Justification

We hypothesized that situations characterized by norm adherence differ from situations involving norm violation. For example, suppose you describe a situation where you won the first prize at a national math tournament. In that case, the situational dimensions analysis may score high on the dimension of “a potentially enjoyable situation.” This dimension indicates the positive feedback accompanied by social norm adherence, specifically the adherence to the norm of success. Another example is a situation of norm violation. Suppose that John is having a job talk. This situation corresponds with Funder’s: “You need to make a good impression.” However, John arrived drunk and tried to punch the boss, which is a situation expressing the dimension: “Someone is breaking rules.”

### Analysis and Results

Each labeled text entered the system and scored according to the one-hundred situational dimensions. Next, the scores (i.e., measurements) were fed as features into another classifier (i.e., Boosting classification) to classify situations labeled as 0 or 1. Using Boosting classification with ten-fold cross-validation, the performance measures appear in Table 2:

**Table 2:** The performance measures on the EmpatheticDialogues dataset.

	0	1
Accuracy	90	90
Precision	87	94
Recall	95	86
Baseline	48	52

Throughout the paper, the baseline always refers to the percentage of labeled cases in the test set.

Fifty-two percent of the situations in the test set are labeled 1, and the rest 0. This is the baseline for a random prediction in which text is labeled as involving the social emotions of shame, pride, etc. One can see that using the 100 scores of the situations’ measurements, the Boosting classification classifier gained 90% accuracy. Moreover, one can see an impressive level of precision and recall.

The most important features, according to their relative influence scores, were the measurements of the dimensions representing items 46 (“Desires could be gratified, for example, food, shopping, sexual opportunities,” Relative influence score = 45.26), 81 (“The situation is physically uncomfortable, for example too hot, too crowded, too cold, etc., Relative influence score = 19.40), 67 (“The situation could arouse positive emotions,” Relative influence score = 17.79), and 31 (“The situation includes small annoyances,” Relative influence score = 4.84).

As the black box of AI is an issue, we used automatic rule discovery to expose simple rules underlying the above classification results. Specifically, we used HeuristicLab’s Optimizer 3.3.1^23^. HeuristicLab’s Optimizer 3.3.16 uses genetic algorithms to perform automatic rule discovery. It starts by generating a population of potential solutions expressed as rules or data structures. Then it applies genetic operators such as crossover and mutation to create new solutions from the existing solutions. This process is repeated until a final population that best meets the problem’s criteria is found. During this process, the most promising rules are stored, which can be used to solve future problems involving the same data and criteria. By the system’s default, 66% of the dataset was used to train the model and the rest for the test. We performed ten folds, each running for 1,000 generations, by examining 99,100 solutions. For the classification task, we used Symbolic Classification analysis^24^ with a maximum symbolic expression length of 5 and a maximum symbolic tree depth of 5. This procedure means we were looking for simple rules through which the situational dimensions may be used to classify the labeled data.

Using the procedure with ten-fold cross-validation, gained 87% accuracy. When examining the ten rules produced by the genetic algorithm, we found that two situational dimensions, representing items 46 and 67, appeared as the only features in 80% of the rules. In the other 20% of the cases, other dimensions/features were involved. It means that the AI (i.e., the genetic algorithm automatic rule discovery system) identified ten rules. In eight out of ten rules, we found that they used only situations 46 or 67. The other two situations included other situational dimensions.

For example, the first rule identified by the system is as follows:

$${\rm{Label}}=\frac{C0\ast 46}{C1\ast 46}$$

where C_0_ = 0.457 and C_1_ = 0.466. To determine whether the text involves something to be proud of or ashamed of, the system measured dimension 46, multiplied it by parameters C_0_ and C_1_, respectively, and divided the first outcome by the second. If the result crosses a threshold, the decision is 1; otherwise, 0. In other words, the system measured the extent to which the text expresses a situation where “Desires could be gratified” and used this measure to determine whether the situation should be labeled 1 or 0. To better explain this rule, we use a concrete example. One of the examples GPT generated for this dimension is “Getting a promotion at work.” Let’s assume that the situation we analyze includes the confession, “I spilled a glass of coffee on my boss’s new suit.” When measuring the situational dimension of “gratified desires,” it is clear that this is a situation where desires, such as getting a promotion, cannot be gratified. If this situational dimension is expressed in the situation to a minor extent, then the conclusion is that this situation does not involve pride. In sum, we can see that the dimensions identified by the classifier and by the Symbolic classification procedure support the face validity of our results and hence the face validity of the items artificially produced by GPT. The features/dimensions used by the machine to classify situations involving pride vs. shame are meaningful to us as human researchers and do not form a black box producing incomprehensible or senseless decisions.

### Experiment 2

#### The dataset

For the second experiment, we used the DailyDialog dataset^25^. The DailyDialog dataset contains a collection of 14,118 dialogues with rich context information in English for modeling conversations. It is designed to support the development of models for understanding and engaging in open-domain conversations. This dataset contains conversations between two speakers, each involving two or more dialogue turns, and covers various topics and day-to-day conversations. This dataset can be used to train chatbot models and generate realistic conversations between two people.

We analyzed the utterances in the dataset and, using a tool for measuring politeness^26^, produced a politeness score ranging from 1 to 5 for each utterance. The politeness score has been converted into two categories of politeness. The first category, “1,” involves cases of impoliteness that scored in the lower 25% percentile of the politeness score (N = 1789). The second category, “3,” involves scores in the upper 25% of cases representing high politeness (N = 1926).

Justification: We hypothesized that situations involving impoliteness differ in their situational representation from situations involving politeness.

#### Analysis and results

We applied the same procedure as the one described in Experiment 1 and used Boosting Classification to predict the binary score of politeness (1 vs 3) based on the 100 situational measurements. The results are presented in Table 3.

In this case, too, we see that the measurements of the situational dimensions provide highly informative features for identifying whether the situation involves politeness or impoliteness.

The most important features, according to their relative influence scores, were the measurements of dimensions 73 (Relative influence score = 36.11), 46 (13.32), 67 (6.44), and 1 (5.78).

These dimensions are:

73: Someone is complimenting or praising you

46: Desires could be gratified (for example, food, shopping, sexual opportunities)

1: The situation is potentially enjoyable

For all these top-rated features, polite utterances scored higher than impolite ones. Using the Mann-Whitney U Test, all differences were found to be statistically significant at p < 0.001, and for dimensions 1 (z = −20.53), 67 (z = −26.52), 46 (−27.528), and 73 (z = −31.73). Therefore, we conclude that politeness is associated with situations where someone compliments or praises, desires could be gratified, and the situation is potentially enjoyable. These items correspond with our understanding of politeness and how it involves what Goffman^27^ described as “Face” or our positive self-image as reflected in interactions with others. Goffman’s idea of “face” directly connects to politeness. When people are polite in their interactions, they tend to maintain the face of others by acting respectfully and not engaging in any conduct that would damage the other person’s image of themselves. Therefore, it is reasonable that when the situational dimension “Someone is complimenting or praising you” scores high, the situation involves politeness rather than impoliteness.

**Table 3:** Performance measures for politeness vs. impoliteness.

	1	3
Accuracy	85	85
Precision	82	88
Recall	88	82
Baseline	48	52

### Experiment 3

We used the Moral Stories dataset^28^ for the third experiment, a “crowd-sourced dataset of structured, branching narratives for the study of grounded, goal-oriented social reasoning” (ibid, p. 698). This dataset contains contexts composed of a norm, a situation, an intention, and two kinds of actions: normative (N = 12000 cases labeled 0) and divergent (N = 12000 cases labeled 1). For example^8^:

Context

Norm: You should not help companies generate pollution.

Situation: Sally is a highly trained biologist.

Intention: Sally wants a prestigious job

Normative path:

Action: Sally goes to work for a company developing technologies for sustainable agriculture.

Consequence: Sally may help fight pollution caused by industrial agriculture.

Divergent path:

Action: Sally goes to work for a company producing chemical fertilizers

Consequence: The fertilizers pollute the land.

Justification: We hypothesized that divergent actions have different situational characteristics than normative actions. For example, suppose the norm is that you should be honest, and the situation is that John joined the police to fight crime. In that case, a normative action may be “John fights crime,” and a divergent non-normative situation may be “John is bribed by the Mafia boss in his neighbourhood.” These are two situations that may differ in their situational dimensions. As seen in the next analysis and results section, dimension 17 was the most important in classifying divergent vs. normative actions. This dimension is grounded in the item: “Someone is attempting to dominate or boss you.” Interestingly, actions successfully classified as non-normative involve a strong situational dimension where someone is trying to dominate the individual and the individual is not acting according to his own free and conscious will. Indeed, many divergent or non-normative actions may be identified as those involving submission to the dominance of forces from drugs and alcohol to vicious leaders or urges.

### Analysis and results

We used the same procedure as described before. The results are presented in Table 4.

Again, the classifier’s performance was far above what could be expected from pure guesses. The most important features, according to their relative influence scores, were the dimensions grounded in items 17, “Someone is attempting to dominate or boss you” (Relative influence score = 29.88), 95, “Prestige” (23.31), 73 “Someone is complimenting or praising you” (14.91), and 98 “Dominance” (6.10).

**Table 4:** Performance measures for the Moral Stories dataset.

	0	1
Accuracy	73	73
Precision	72	74
Recall	74	73
Baseline	49	51

### Experiment 4

The SOCIAL-CHEM-101 dataset^29^ was used for the final analysis. This dataset contains 104k real-life situations obtained and processed through crowdsourcing. It contains labels for actions such as running the blender at 5 am, labeled as “legal” or “illegal.” The percentage of illegal actions was very small, so N = 5934 unique actions labeled as illegal were identified and matched with an equal number of actions labeled as legal (N = 5934).

Justification: We hypothesized that legal and illegal actions differ concerning their situational dimensions. For instance, dimension 53 represents a situation where “someone is breaking the rule.” It is hypothesized that automatically measuring this dimension can be used to decide whether the described action is legal or illegal.

### Analysis and results

We used Boosting Classification to predict the binary score of legal vs. illegal actions (0 vs 1, respectively). We used the 100 scores as features and applied a ten-fold cross-validation procedure. The results are presented in Table 5.

**Table 5:** Performance measures for the Social Chemistry dataset. The base is line is 50%.

	0	1
Accuracy	84	84
Precision	82	85
Recall	86	81

In this experiment, the accuracy is far above what should have been expected from a random guess based on the baseline of the two categories. Moreover, the most important features, according to their relative influence scores, were the measurements of dimensions 53 (Relative influence score = 51.09), 57 (Relative influence score = 9.33), 45 (7.95), and 15 (7.51). Dimension 53, as mentioned in the justification section, is “someone is breaking the rule.” This situational dimension can differentiate between legal and illegal actions when correctly measured Table 5.

## Data Availability

The code for the SetFit is available here: 10.6084/m9.figshare.23254856.v2^17^.
